# Prevalence of *Rickettsia* species in ticks including identification of unknown species in two regions in Kazakhstan

**DOI:** 10.1186/s13071-019-3440-9

**Published:** 2019-05-03

**Authors:** Nurkeldi Turebekov, Karlygash Abdiyeva, Ravilya Yegemberdiyeva, Andrey Dmitrovsky, Lyazzat Yeraliyeva, Zhanna Shapiyeva, Aday Amirbekov, Aksoltan Oradova, Zulfiya Kachiyeva, Lyazzat Ziyadina, Michael Hoelscher, Guenter Froeschl, Gerhard Dobler, Josua Zinner, Stefan Frey, Sandra Essbauer

**Affiliations:** 1 Center for International Health, Ludwig-Maximilians-University, Munich, Germany; 2 Central Reference Laboratory, Kazakh Scientific Center for Quarantine and Zoonotic Diseases, Almaty, Kazakhstan; 3 Department of Infectious and Tropical Diseases, Kazakh National Medical University, Almaty, Kazakhstan; 4 Department of Children’s Infectious Diseases, Kazakh National Medical University, Almaty, Kazakhstan; 5 Scientific Practical Center of Sanitary Epidemiological Expertise and Monitoring, Almaty, Kazakhstan; 6 Research Institute of Applied and Fundamental Medicine, Kazakh National Medical University, Almaty, Kazakhstan; 7 Division of Infectious Diseases and Tropical Medicine, University Hospital, Ludwig-Maxmilians-University, German Center for Infection Research, Munich Partner site, Munich, Germany; 8 Department Virology & Rickettsiology, Bundeswehr Institute of Microbiology, German Center for Infection Research, Munich Partner site, Munich, Germany

**Keywords:** Ticks, *Rickettsia slovaca*, *Rickettsia raoultii*, DNA isolation, Almaty region, Kyzylorda region, Kazakhstan

## Abstract

**Background:**

Over 60 years ago clinical patterns resembling tick-borne rickettsioses have been described for the first time in Kazakhstan. Since 1995 the incidence of clinical cases of tick-borne rickettsioses in humans seems to be rising but studies on epidemiological data regarding the occurring etiological agents, tick vector species, prevalence and distribution throughout Kazakhstan are still scarce to date. The aim of the study was molecular investigation of ticks for spotted-fever group rickettsiae in the endemic Kyzylorda region and the so far considered as non-endemic Almaty region. A total of 2341 ticks was collected in the two regions in Kazakhstan and sorted in 501 pools: *Ixodes persulcatu*s (243); *Dermacentor marginatus* (129); *Haemaphysalis punctata* (104); *Hyalomma asiaticum* (17); *Dermacentor reticulatus* (3); and *Rhipicephalus turanicus* (5). Pools were tested for *Rickettsia* spp. using real-time PCR. For positive samples multilocus sequence typing (MLST) was performed.

**Results:**

The calculated minimum infection rate (MIR) for rickettsiae in the investigated ticks in Almaty region varied between 0.4–15.1% and 12.6–22.7% in the Kyzylorda region. At least four different *Rickettsia* species were identified in the two selected regions of Kazakhstan. Two of these are already known to science: *Rickettsia raoultii* and *R. slovaca*, the latter being reported for the first time in Almaty region One new form, “*Candidatus* R. yenbekshikazakhensis”, was described by MLST of six gene fragments in Almaty region and one new genotype, “genotype R. talgarensis” was detected using three gene fragments.

**Conclusions:**

Kazakh physicians should be aware of rickettsioses after tick bites in both regions studied. Both, *R. raoultii* and *R. slovaca* should be included in the diagnostics. The role for human diseases has further to be investigated for the newly described rickettsiae, “*Candidatus* R. yenbekshikazakhensis” and “Genotype R. talgarensis”.

## Background

Bacteria in the genus *Rickettsia* are arthropod-transmitted pathogens of vertebrates [1]. Rickettsiae are intracellular parasites, and are symbionts in the broad sense as these have close relationships with their hosts. They are the causative agents of numerous diseases of humans [2] which can occur from subclinical to severe forms [1, 3]. According to recent data, *Rickettsia* spp. that cause infections in humans are divided into two major groups: the typhus group (*Rickettsia prowazekii* and *Rickettsia typhii)* and the spotted fever group (SFG) (*Rickettsia rickettsii*, *Rickettsia slovaca*, *Rickettsia sibirica*, *Rickettsia raoultii*, *Rickettsia conorii*, *Rickettsia peacockii*, *Rickettsia honei*, *Rickettsia japonica*, *Rickettsia montanensis*, *Rickettsia massiliae*, *Rickettsia ripicephali*, *Rickettsia amblyommii*, *Rickettsia africae*, *Rickettsia parkeri*, *Rickettsia heilongjiangensis*, *Rickettsia phillipi*). The major typhus group includes the typhus group itself and the “ancestral” group with *R. bellii* subgroup and *R. canadensis* subgroup. The major spotted fever group consists of the “classical” spotted fever group (*R. rickettsia* subgroup, *R. conorii* subgroup, *R. australis* subgroup) and two transitional groups, *R. felis* group and *R. akari* group. Rickettsiae are widespread among arthropods including lice, fleas and most species of ixodid ticks [4–6].

The knowledge on the tick-associated rickettsiae and their significance of inducing human diseases has been considerably enhanced in the past three decades The main reason for progress is that molecular methods such as multilocus sequence typing (MLST) or next-generation sequencing have helped to identify new and previously recognized rickettsiae in ticks [7]. MLST led to the description of several new “Candidatus” *Rickettsia* species by describing at least four or five gene fragments or new *Rickettsia* genotypes if less than four sequences are characterized [4, 8–10].

The clinical pictures of human cases of tick-born rickettsioses were first described in Kazakhstan during expeditions to Almaty region in 1949–1951 [11]. A few years later, clinical pictures of tick-borne rickettsioses were described further in five districts i.e. South Kazakhstan, West Kazakhstan, Pavlodar, North Kazakhstan and Akmola regions [12]. The causative agent of the North Asian tick-borne rickettsiosis (*R. sibirica*) was first described and isolated in 1961 by intra-abdominal infection of guinea pig males with homogenates containing *Dermacentor marginatus* and *Haemaphysalis punctata* ticks, which were collected in Yenbekshikazakh district of Almaty region [13]. Since 1995, clinical case definition criteria and a complement fixation test (CFT) with *R. sibirica* are used in Kazakhstan for diagnostics and consequently official registration of tick-borne rickettsiosis cases in humans. Currently, annual data exist for four regions in Kazakhstan (North Kazakhstan, Pavlodar, East Kazakhstan and Kyzylorda), which are currently considered as endemic regions for tick-borne rickettsioses. According to available statistical data, in total 3904 human cases of tick-borne rickettsiosis were officially registered in Kazakhstan from 1995 to 2016. In this period the incidence rate of this disease increased from 0.41 to 0.87 (per 100,000 inhabitants per year). The biggest increase was observed during this period in Kyzylorda region (incidence of 1.64–11.1 per 100,000 inhabitants per year) and Pavlodar region (incidence of 1.07–7.0 per 100,000 inhabitants per year). According to the currently available data, the Kyzylorda region is supposed to be an endemic area for tick-borne rickettsioses in Kazakhstan [14].

There exist some data on *Rickettsia* species that might circulate in the endemic regions of Kazakhstan. *Rickettsia raoultii* was reported in *Dermacentor* spp. and *Ixodes* spp. in three regions (Kyzylorda, Karaganda and East Kazakhstan) [15–20] and *R. conorii caspia*, *R. raoultii* and *R. aeschlimannii* were detected in ticks collected in the western, northern and central regions of Kazakhstan [21]. *Rickettsia aeschlimannii* was reported in *Haemaphysalis punctata* originating from Almaty region [17, 18]. Recently, Hay et al. (2016) [22] demonstrated the presence of *R. conorii caspia* in ticks engorged on four-striped grass rats (*Rhabdomys pumilio*) collected in the West Kazakhstan region. In 2017, *Rickettsia asembonensis* and *Rickettsia felis/*“*Candidatus* Rickettsia senegalensis” were detected in fleas collected in Almaty region [22, 23].

So far data concerning *Rickettsia* spp. from the spotted fever group circulating in the Almaty region are limited and there are no registered epidemiological data on human infections from this region [24]. Currently, there are still large gaps regarding the knowledge on circulating *Rickettsia* species in ticks and their geographical distribution in Kazakhstan. Here, we present data of a molecular study of ticks for SFG rickettsiae in two regions, the Almaty region, which is considered so far non-endemic but remains the most densely populated region in Kazakhstan, and in the endemic Kyzylorda region.

## Methods

### Tick sampling

Ticks were collected by flagging the vegetation in three districts of Almaty region (Talgar, Yeskeldy and Yenbekshikazakh districts) and Kyzylorda region (Syrdarya, Shyeli and Zhanakorgan districts), Kazakhstan, in May-June 2015.

The sampling sites of the ticks of Almaty region have the following characteristics: Yeskeldi district (44°54′12″N, 78°29′42″E) with Tekeli city (44°49′48″N, 78°49′26″E) is located in Almaty region adjacent to the Peopleʼs Republic of China and is characterized by coniferous forests and open steppe vegetation. The region is mountainous with an altitude of 1400–2200 m above sea level (masl). More than 40% of the area is covered by forest, and the remaining parts constitute of pasture and agricultural land. Animal husbandry is practiced widely. The average annual precipitation has been reported as 250–300 mm [25]. Talgar district (43°18′55″N, 77°14′35″E) with Talgar city (43°18′0″N, 77°14′0″E) is 40 km away from Almaty city and comprises forested taiga, forested steppe and arid fields, the latter mainly covered by gramineous plants. Nearly 20% of its northern part is at an altitude of 1800–2400 masl. The annual precipitation is 200–300 mm [25]. Yenbekshikazakh district (43°21′0″N, 77°28′0″E) with Yesyk city (43°21′0″N, 77°28′0″E) has an altitude of 560–1300 masl and offers areas of maritime climate in summer and very cold temperatures (from -25 °C down to -50 °C) during winter. Average precipitation is reported as 200–700 mm/year. Plain steppe and meadows dominate most parts of this area [25].

In the Kyzylorda region, ticks were collected in three districts: Syrdarya (45°34′12″N, 65°36′0″E), Shyeli (44°10′0″N, 66°44′0″E) and Zhanakorgan (43°56′24″N, 67°13′12″E). Kyzylorda region (45°0′0″N, 64°0′0″E) is located in the south-western part of Kazakhstan, to the east of the Aral Sea in the lower reaches of the River Syrdarya, mainly within the Turan Lowland (altitude of 50–200 masl). The region borders the neighboring country Uzbekistan, as well as three other Kazakh regions: Aktobe region (to the west), Karaganda region (to the north), and South Kazakhstan (to the east). The climate is rather continental and extremely arid with prolonged hot and dry summers and with a comparatively warm, short and moderate winter. The amount of precipitation in the north-west near the Aral Sea coast is about 100 mm (the lowest in Kazakhstan) and up to 175 mm in the southeast, in the foothills of Karatau Mountain. A significant part of the region is occupied by sands, almost devoid of vegetation [26].

### Sample preparation

The collected ticks were stored at -20 °C until further study. The laboratory study was conducted in batches. After thawing, all field ticks have been sorted by genus, species, stage and sex following the official guidelines for tick specification in Kazakhstan [27–30]. Next, the ticks were grouped into pools by genus, species, stage and sex (with a maximum of 5 adult ticks in a pool). Each pool has been homogenized using the TissueLyser II instrument, after adding ceramic granules and 1 ml medium Dulbecco’s Modified Eagle Medium (DMEM) (BioloT, Saint-Petersburg, Russia) to each tube. Following Kazakh guidelines for biosafety and biosecurity, aliquots containing tick homogenates were inactivated in a water bath at 56 °C for 30 min, before DNA extraction. DNA was extracted from 200 μl tick homogenates using the QIAamp DNA Mini Kit (Qiagen, Hilden, Germany), according to the manufacturer’s instructions.

### PCR and species identification of rickettsiae

All PCRs were conducted in a three-room-regime. The presence of rickettsial DNA was determined by a real-time PCR assay targeting the pan-rickettsial citrate synthase gene using the primers PanRick_*gltA*_2_for (5′-ATA GGA CAA CCG TTT ATT T-3′), PanRick_*gltA*_2_rev (5′-CAA ACA TCA TAT GCA GAA A-3′), PanRick_*gltA*_2_taq (5′-6FAM-CCT GAT AAT TCG TTA GAT TTT ACC G-DB-3′) and Uracil-DNA-glycosylase (UDG) in order to eliminate carry-over contamination [31, 32] in a Rotor-Gene Q (Qiagen) machine.

In all samples yielding a positive pan-rickettsial citrate synthase gene signal, a multilocus sequence typing (MLST) targeting six gene fragments (partial fragments of *ompB*, *ompA*IV, 23S-5S interspacer region, *16S*, *sca4*, *gltA*) was conducted for *Rickettsia* species identification [31–34]. First, for all these samples the partial outer membrane protein B (*ompB*) gene (RR 120–2788, cRR 120–3599) was amplified following published protocols [34]. Secondly, for the samples with positive sequences (depending on the sequence result) five additional fragments were investigated using previously published primers [31–38]: *ompA*IV (RR 190–5125, cRR 190–6013), 23S-5S interspacer region (23s for, 23s rev), *16S* (Ric, Ric RT), *sca4* (Rsca4_1707f, Rsca4_2837r) and partial *gltA* (Rh314, Rh654) [31–34].

Master mix solution for 23S-5S interspacer region PCR (50 µl including 5 µl DNA) were prepared with 0.2 mM dNTP Mix (Thermofisher-Invitrogen, Schwerte, Germany), 0.5 µM of each primer (23s for, 23s rev), 1.5 U Platinum^®^ *Taq* DNA Polymerase High Fidelity (Thermofisher-Invitrogen), 1× PCR buffer (Thermofisher) and 2.0 mM MgSO_4_ (Thermofisher). The initial denaturation was performed for 3 min at 95 °C, 45 cycles of amplification each starting with denaturation for 20 s at 95 °C, followed by annealing for 30 s at 57 °C and elongation for 60 s at 68 °C, and a final elongation step at 68 °C for 10 min [35, 39].

Gene D (*sca4*) sequences were amplified with 0.2 mM dNTP Mix (Thermofisher-Invitrogen), 0.1 µM of each primer (Rsca4_1707f, Rsca4_2837r), 1.0 U Platinum^®^ Taq DNA Polymerase High Fidelity (Thermofisher-Invitrogen), 1× PCR buffer, 3 mM MgSO_4_ and 2 µl DNA in a final reaction volume of 50 µl. After an initial denaturation for 3 min at 95 °C, 40 cycles with denaturation for 30 s at 95 °C, annealing for 35 s at 53 °C and elongation for 90 s at 68 °C, and a final extension step at 68 °C for 7 min [37, 39].

Partial *16S* sequences were amplified using 0.2 mM dNTP Mix (Thermofisher-Invitrogen), 0.5 µM of each primer (Ric, Ric RT) with 1.5 U Platinum^®^ Taq DNA Polymerase High Fidelity (Thermofisher-Invitrogen), 1× PCR buffer, 2.5 mM MgSO_4_ and 5 µl DNA in a final reaction volume of 50 µl. After an initial denaturation for 3 min at 95 °C, 45 cycles with denaturation for 30 s at 94 °C, annealing for 30 s at 63 °C, and elongation for 120 s at 68 °C were performed, followed by a final extension step at 68 °C for 7 min [36, 39].

The partial *gltA* gene was amplified with 0.2 mM dNTP Mix (Thermofisher- Invitrogen), 0.5 µM of each primer (Rh314, Rh654), 1.0 Unit Platinum^®^ Taq DNA Polymerase High Fidelity (Thermofisher-Invitrogen), 1× PCR buffer, 2 mM MgSO_4_ and 5 µl DNA in a final reaction volume of 50 µl. After an initial denaturation for 2 min at 94 °C, 45 cycles with denaturation for 20 s at 94 °C, annealing for 30 s at 54 °C and elongation for 60 s at 68 °C were conducted, followed by a final extension step at 68 °C for 5 min [38, 39].

PCR products were visualized in a 1.5% agarose gel and purified using the QIAquick PCR Purification Kit (Qiagen) according to the manufacturer’s recommendations. PCR product sequencing was carried out using the ABI Prism BigDye Terminator v3.1 Cycle Sequencing Kit (Applied Biosystems) and 3500xl Genetic Analyzer (Hitachi, Japan) with the primers used for the PCR amplifications. pGEM-3Zf(+) control template was used as a sequencing control. Quantification of the PCR products was performed on a Fluorometer Qubit 2.0 (Invitrogen, USA). Sequence analyses were carried out with Chromas Lite 2.01 [40] and Bioedit 7.2.5. [41]. Obtained sequences were compared with sequences from GenBank using BLAST 2.2.32 [42, 43].

Phylogenetic trees were constructed using the Maximum Likelihood method based on the Tamura 3-parameter model [44] with the software package MEGA 6 [45]. The percentage of trees in which the associated taxa clustered together is shown next to the branches. Initial trees for the heuristic search were obtained automatically by applying Neighbor-Join and BioNJ algorithms to a matrix of pairwise distances estimated using the Maximum Composite Likelihood (MCL) approach, and then selecting the topology with superior log-likelihood value. The tree is drawn to scale, with branch lengths measured in the number of substitutions per site.

The binomial (Clopper-Pearson) ‘exactʼ method based on the beta distribution was used for the calculation of 95% confidence intervals (CI).

The minimum infection rate (MIR) was calculated as the ratio of the number of positive tick pools to the total number of ticks of the same species.

## Results

**Fig. 1:**
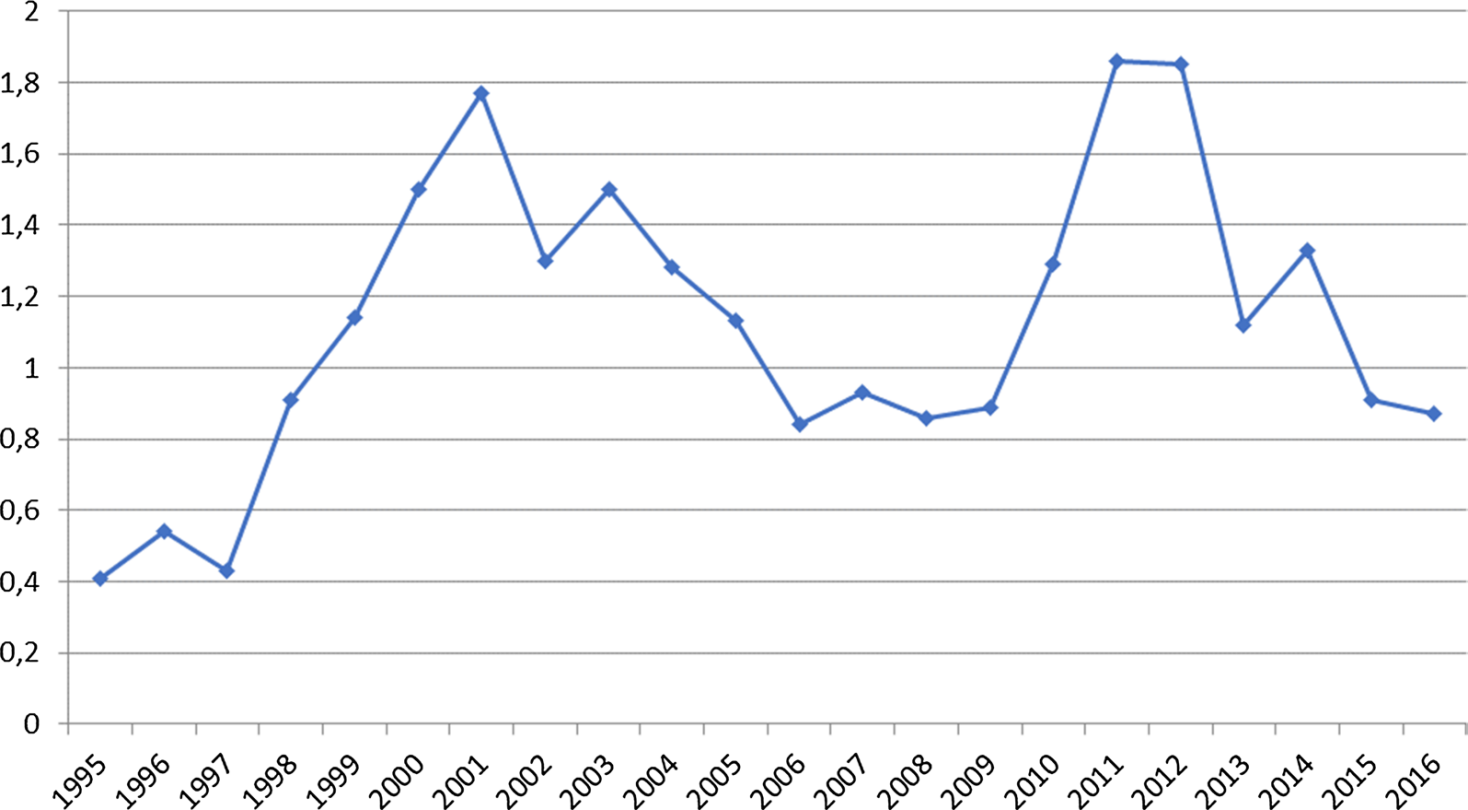
Incidence of tick-borne rickettsiosis in Kazakhstan based on complement fixation test (modified after [14])

Six tick species collected from 6 districts of Almaty and Kyzylorda regions in Kazakhstan were studied: *Ixodes persulcatus* (*n* = 1193; 243 pools); *Dermacentor marginatus* (*n* = 578; 129 pools); *Haemaphysalis punctata* (*n* = 470; 104 pools); *Hyalomma asiaticum* (*n* = 77; 17 pools); *Dermacentor reticulatus* (*n* = 14; 3 pools); and *Rhipicephalus turanicus* (*n* = 9; 5 pools). *Ixodes persulcatus*, *D. marginatus* and *H. punctata* were the most abundant tick species collected. Four tick species out of six (*I. persulcatus*, *H. punctata*, *D. marginatus* and *D. reticulatus*) were detected in Almaty region, with three of them (*I. persulcatus*, *H. punctata* and *D. reticulatus*) found only in this region. Three tick species out of six (*Hy. asiaticum*, *Rh. turanicus*, *D. marginatus*) were collected in Kyzylorda region, with two of them (*Hy. asiaticum* and *Rh. turanicus)* found only from this region. *Dermacentor marginatus* was collected more often in Kyzylorda region than in Almaty region (Table 1). Figure 1 provides an overview of collection sites and tick species studied.

**Table 1:** Summary data for collected tick species originating from two regions in Kazakhstan

Locality	No. of ticks	No. of pools	*I. persulcatus*		*H. punctata*		*D. marginatus*		*D. reticulatus*		*H. asiaticum*		*R. turanicus*	
			*n*	Pools	*n*	Pools	*n*	Pools	*n*	Pools	*n*	Pools	*n*	Pools
Almaty region														
Talgar	505	104	504	103	1	1	0	0	0	0	0	0	0	0
Yeskeldy	709	148	610	123	25	7	60	15	1	3	0	0	0	0
Yenbekshikazakh	523	113	79	17	444	96	0	0	40	0	0	0	0	0
Total	1737	365	1193	243	470	104	60	15	14	3	0	0	0	0
Kyzylorda region														
Syrdarya	203	46	0	0	0	0	203	46	0	0	0	0	0	0
Shieli	202	46	0	0	0	0	199	43	0	0	0	0	3	3
Zhanakorgan	199	44	0	0	0	0	116	25	0	0	77	17	6	2
Total	604	136	0	0	0	0	518	114	0	0	77	17	9	5
Grand total	2341	501	1193	243	470	104	578	129	14	3	77	17	9	5

The overall prevalence as per minimum infection rate (MIR) of rickettsial DNA in the tick species and in the collecting localities (Almaty and Kyzylorda regions) was 42.3% (212/501; 95% CI: 37.9–46.8%). The largest number of the *Rickettsia* partial *gltA* real-time PCR-positive tick pools was determined in *Dermacentor* spp. (128/132; 97.0%; 95% CI: 92.4–99.2%) and *H. punctata* (79/104; 76.0%; 95% CI: 66.6–83.8%) (Table 1) collected from three selected districts of Kyzylorda region (*Rickettsia* prevalence 56.8–100%) and in Yenbekshikazakh district (79/113; 69.9%; 95% CI: 60.6–78.2%) of Almaty region (Table 2). The smallest number of the *Rickettsia* partial *gltA* real-time PCR-positive tick pools was found in *I. persulcatus* (3/243;1.2%; 95% CI: 0.3–3.6) and *H. asiaticum* (1/17; 5.9%; 95% CI: 0.2–28.7%) (Table 1) collected from Almaty region (*Rickettsia* prevalence 1.9–11.5%) and in Zhanakorgan district (25/44; 56.8%; 95% CI: 41–71.7%) of Kyzylorda region (Table 3). All *Rh. turanicus* from Zhanakorgan district of Kyzylorda region were negative in the screening real-time PCR (Table 1).

**Table 2:** Distribution of rickettsial DNA in the collected tick species

Tick species	*gltA* real-timePCR positive (%)^a^	No. of ticks	MIR (%)^b^
*Ixodes persulcatus*	1.2 (3/243)	1193	0.3
*Haemaphysalis punctata*	76.9 (80/104)	470	17.0
*Dermacentor marginatus*	96.9 (125/129)	578	21.6
*Dermacentor reticulatus*	100 (3/3)	14	21.4
*Hyalomma asiaticum*	5.9 (1/17)	77	1.3
*Rhipicephalus turanicus*	0 (0/5)	9	0
Total	42.3 (212/501)	2341	9.1

^a^Percent positive (number of *gltA* positive pools/total number of pools)

^b^MIR = number of positive pools/ number of tested ticks

**Table 3:** Distribution of rickettsial DNA in the collecting localities

Locality	*gltA* rtPCR-positive (%)^a^	No. of ticks	MIR (%)^b^
Almaty region			
Talgar	1.9 (2/104)	505	0.4
Yeskeldy (Tekeli city)	11.5 (17/148)	709	2.4
Yenbekshikazakh	69.9 (79/113)	523	15.1
Kyzylorda region			
Syrdarya	100.0 (46/46)	203	22.7
Shieli	93.5 (43/46)	202	21.3
Zhanakorgan	56.8 (25/44)	199	12.6
Total	42.3 (212/501)	2341	9.1

^a^Percent positive (number of *gltA* positive pools/total number of pools)

^b^MIR = number of positive pools/ number of tested ticks

Moreover, the MIR was calculated for each tick species and for both selected regions in this study (Tables 1 and 2). A high MIR of rickettsiae was detected in *Dermacentor* (MIR = 21.4–21.6%) and *Haemaphysalis* (MIR = 17.0%) ticks (Table 2) collected from Yenbekshikazakh district (Almaty region, MIR = 15.1%) and from the three districts of Kyzylorda region (MIR = 12.6–22.7%) (Table 3).

**Fig. 2:**
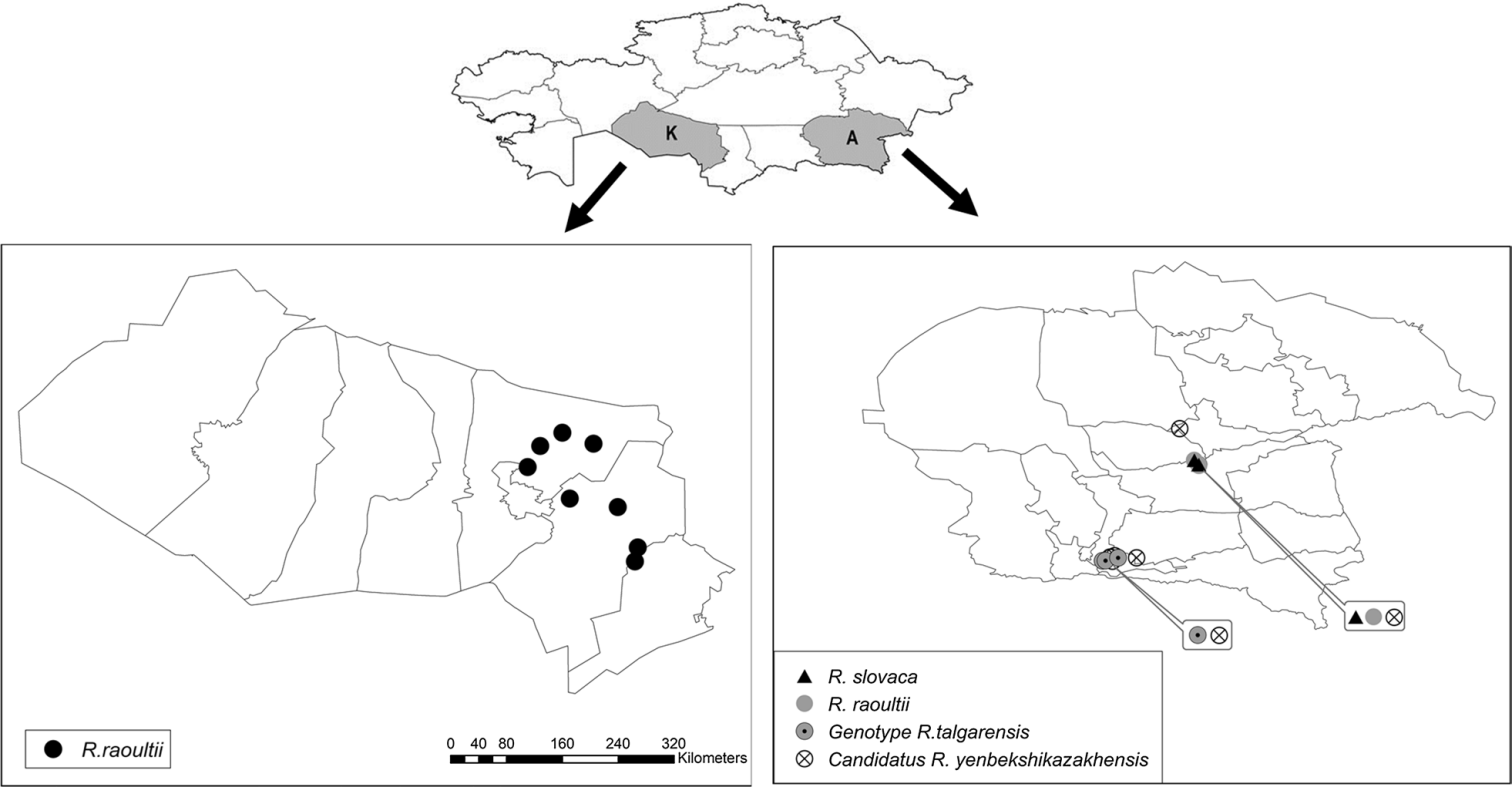
Distribution of the species of *Rickettsia* detected in the Almaty and Kyzylorda regions. The small map on top shows the geographical location of the two regions in Kazakhstan

**Fig. 3:**
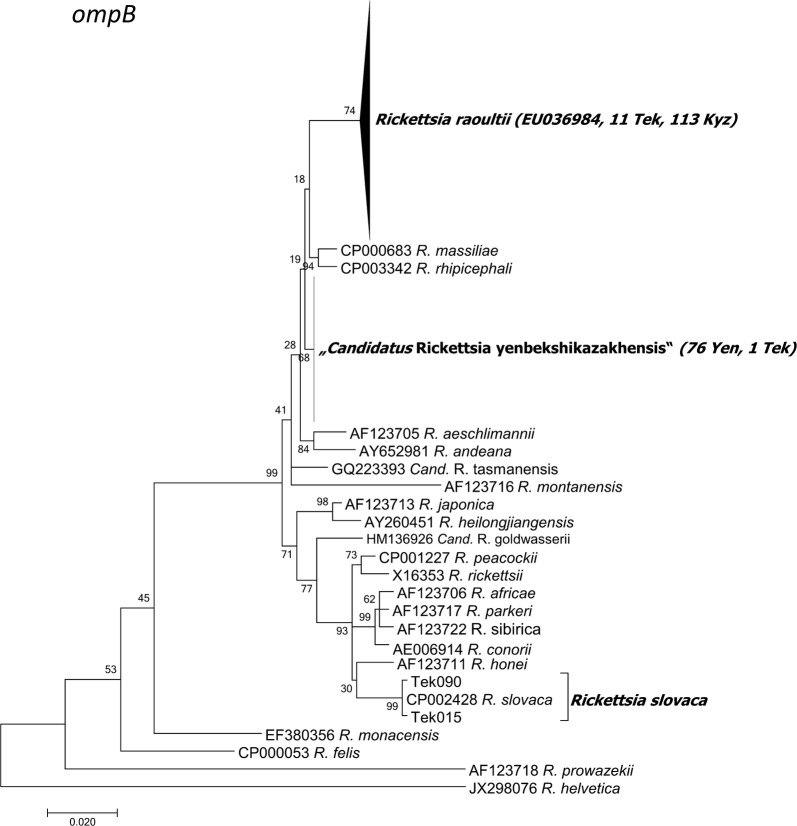
Maximum Likelihood phylogenetic tree based on 226 partial *ompB* DNA sequences, with 203 sequences originating from amplificates from Kazakh tick DNA and 23 from the GenBank database. 124 sequences from Kazakh ticks were 100% identical to *R. raoultii*, two were 100% identical to *R. slovaca*, and 77 sequences formed a new cluster “*Candidatus* Rickettsia yenbekshikazakhensis” (76 sequences from Yenbekshikazakh district, 1 from Yeskeldy district-Tekeli city). The tree with the highest log-likelihood (-3541.6714) is shown. There were a total of 806 positions in the final dataset

**Fig. 4:**
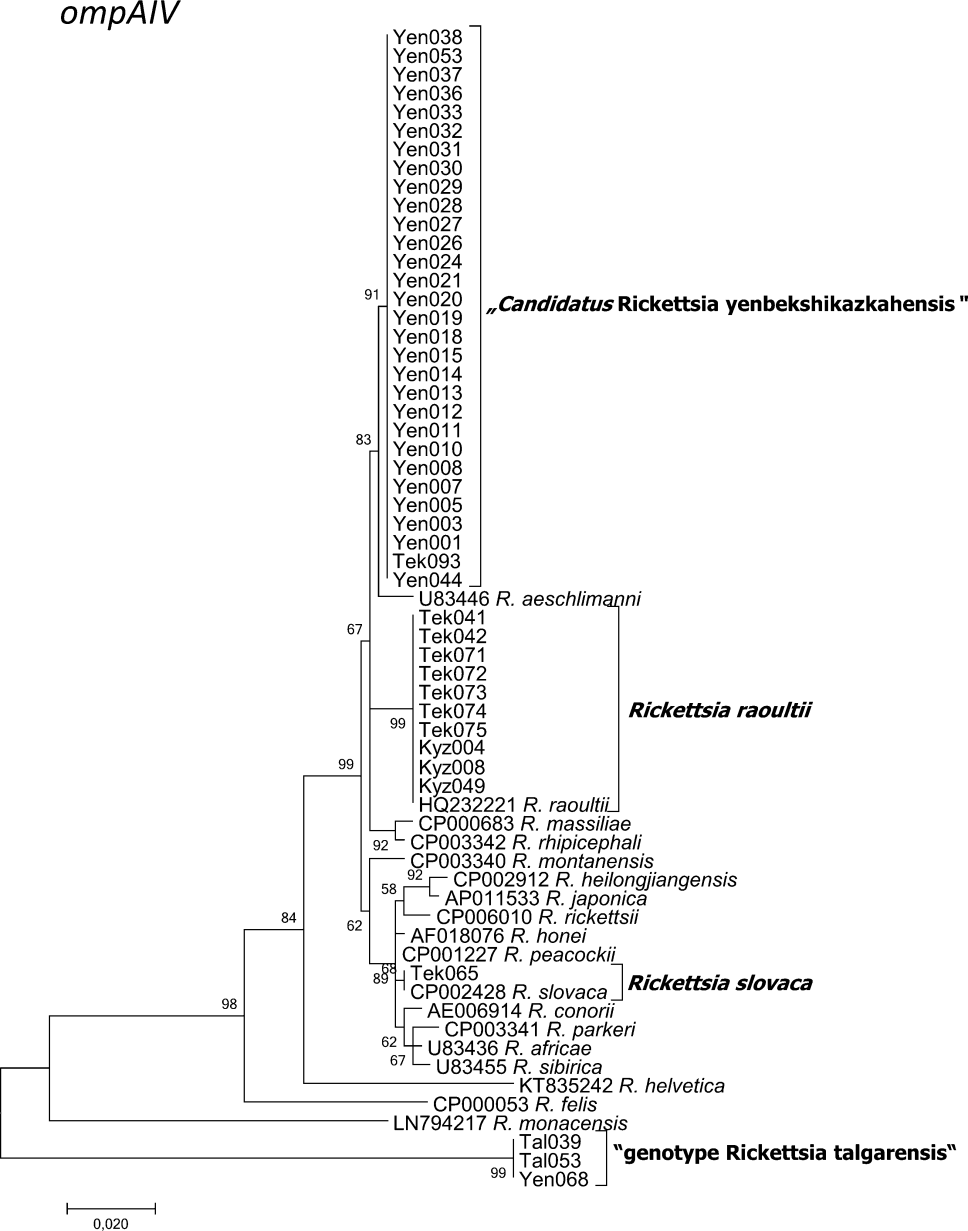
Maximum Likelihood phylogenetic tree based on 62 partial *ompA*IV sequences, with 44 sequences originating from amplificates from Kazakh tick DNA and 20 from the GenBank database. 10 sequences from Kazakh ticks were 100% identical to *R. raoultii*, one sequence was identical to *R. slovaca*. 30 sequences formed a new cluster “*Candidatus* Rickettsia yenbekshikazakhensis” (29 sequences from Yenbekshikazakh district and 1 from Yeskeldy district around Tekeli city) and three a new cluster “genotype Rickettsia talgarensis” (1 sequence from Yenbekshikazakh district, 2 from Yeskeldy district-Tekeli city). There were a total of 864 positions in the final dataset. The tree with the highest log-likelihood (-1803.5066) is shown

**Fig. 5:**
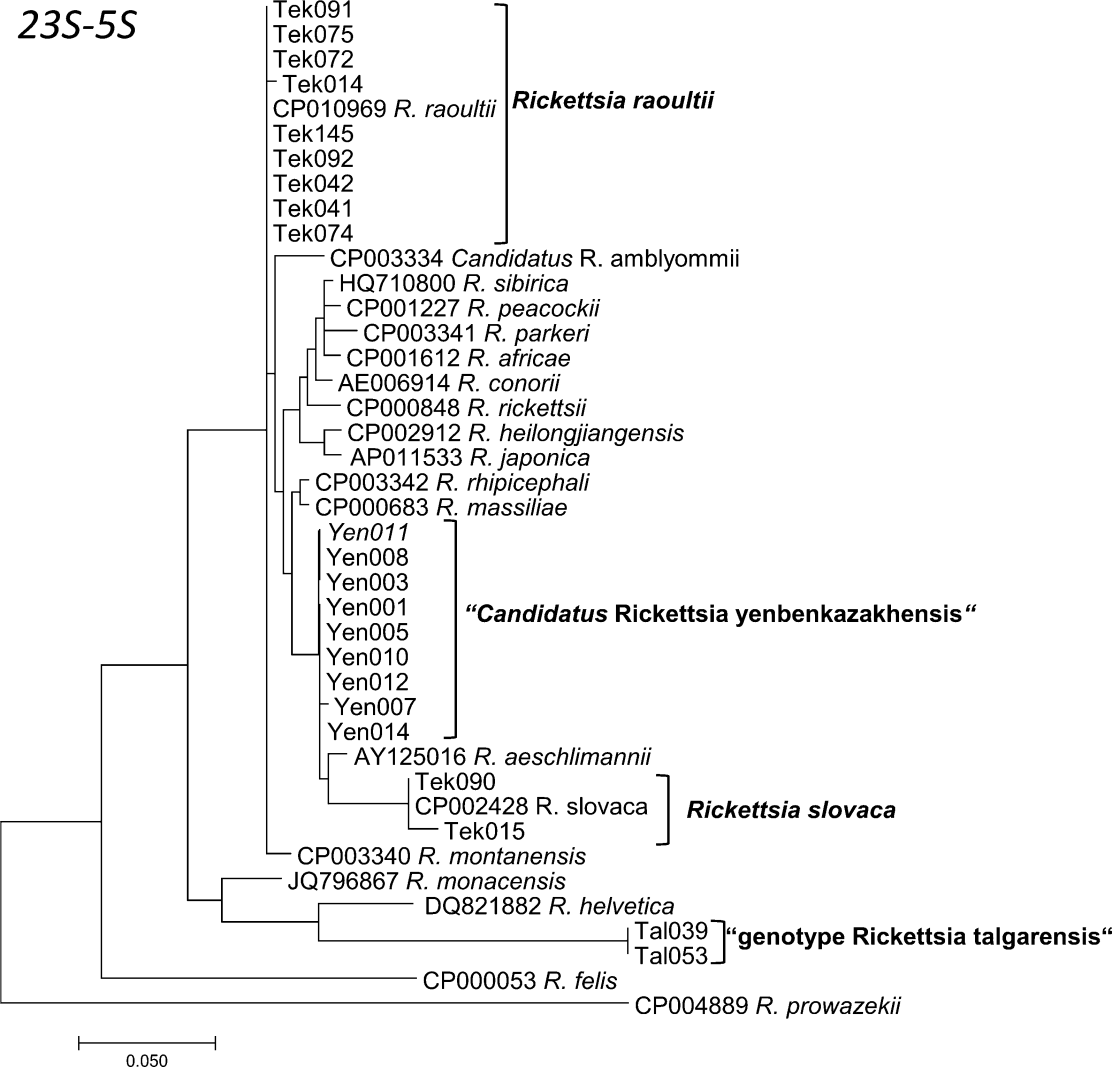
Maximum Likelihood phylogenetic tree based on 40 partial 23S-5S sequences, with 22 sequences originating from Kazakh ticks and 18 from GenBank. Nine sequences from Kazakh ticks were 100% identical to *R. raoultii*, two sequences were identical to *R. slovaca*. Nine sequences Yenbekshikazakh district formed a new cluster “*Candidatus* Rickettsia yenbekshikazakhensis“. There were a total of 367 positions in the final dataset. The tree with the highest log-likelihood (-1572.3294) is shown

**Fig. 6:**
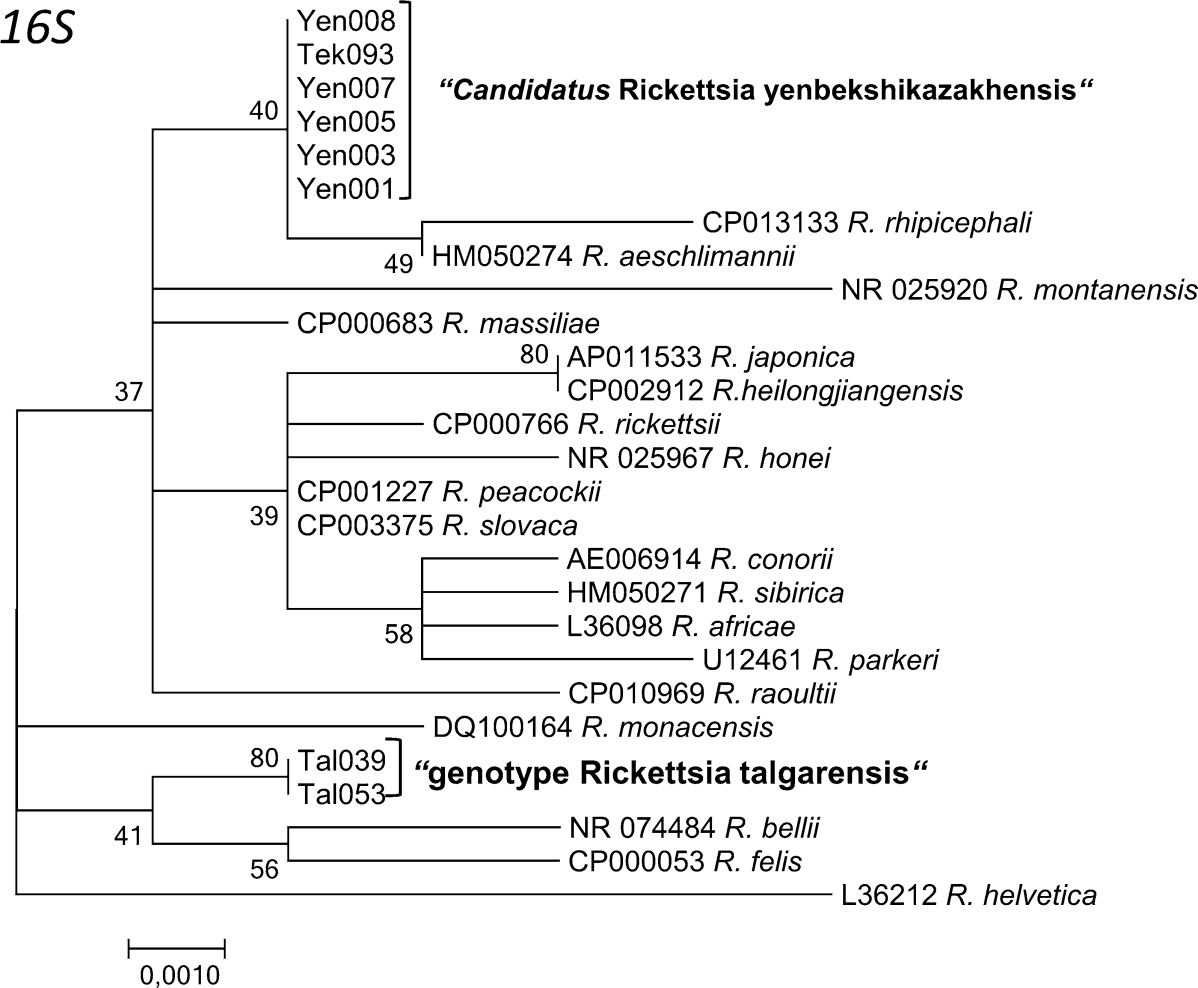
Maximum Likelihood phylogenetic tree based on partial 27 partial *16S* sequences, with 8 sequences originating from Kazakh ticks and 19 from GenBank. Six sequences formed a new cluster “*Candidatus* Rickettsia yenbekshikazakhensis” (5 sequences from Yenbekshikazakh district, 1 from Yeskeldy district-Tekeli city) and two sequences from DNA of ticks from Tekeli the new cluster “genotype Rickettsia talgarensis”. There were a total of 717 positions in the final dataset. The tree with the highest log-likelihood (-1287.3794) is shown

**Fig. 7:**
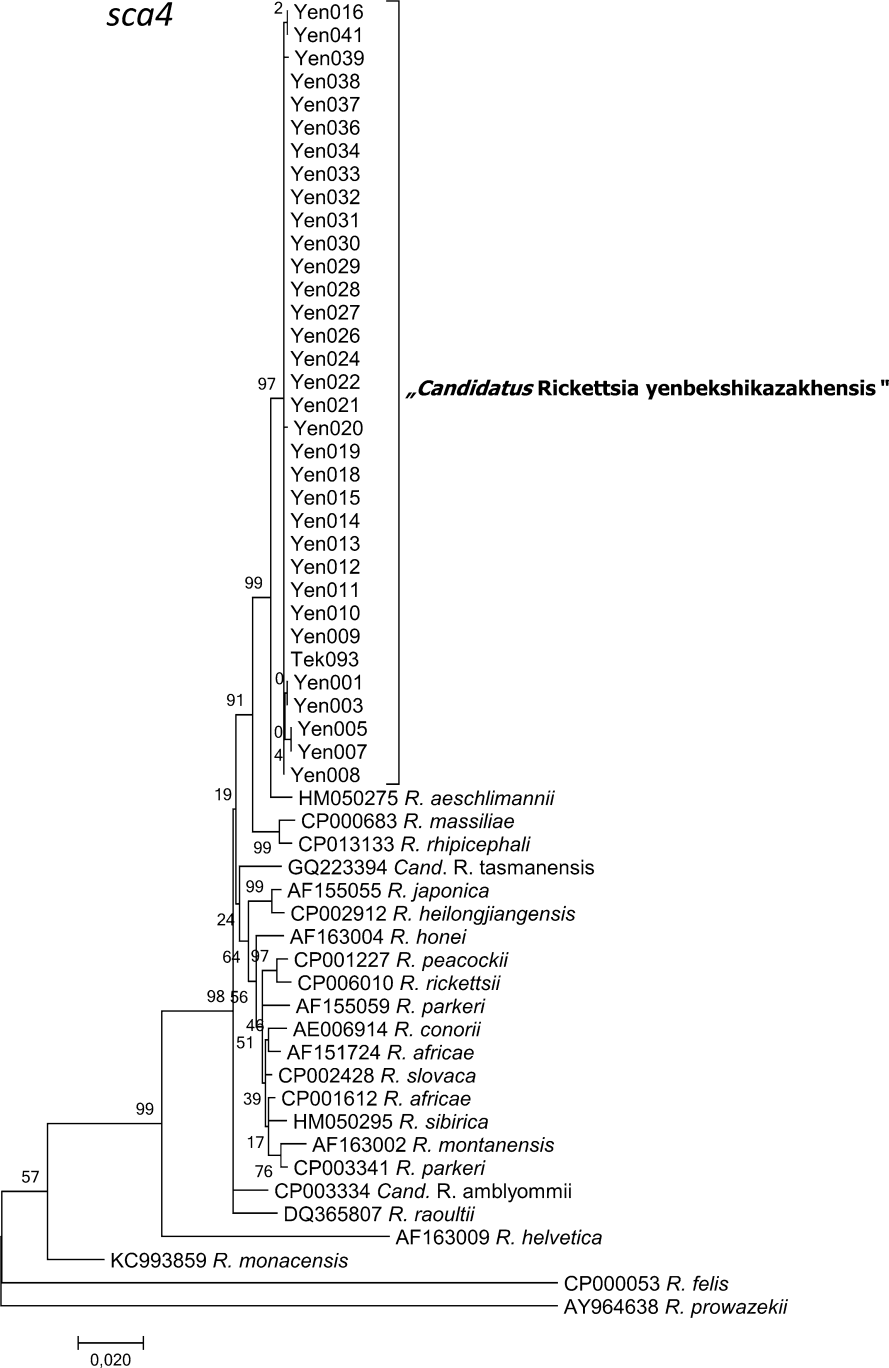
Maximum Likelihood phylogenetic tree based on f partial 57 *sca4* sequences with 34 sequences originating from Kazakh tick DNAs (33 from Yenbekshikazakh district, 1 from Yeskeldy district-Tekeli city) and 23 from GenBank. There were a total of 1.115 positions in the final dataset. The tree with the highest log-likelihood (-4809.7101) is shown

**Fig. 8:**
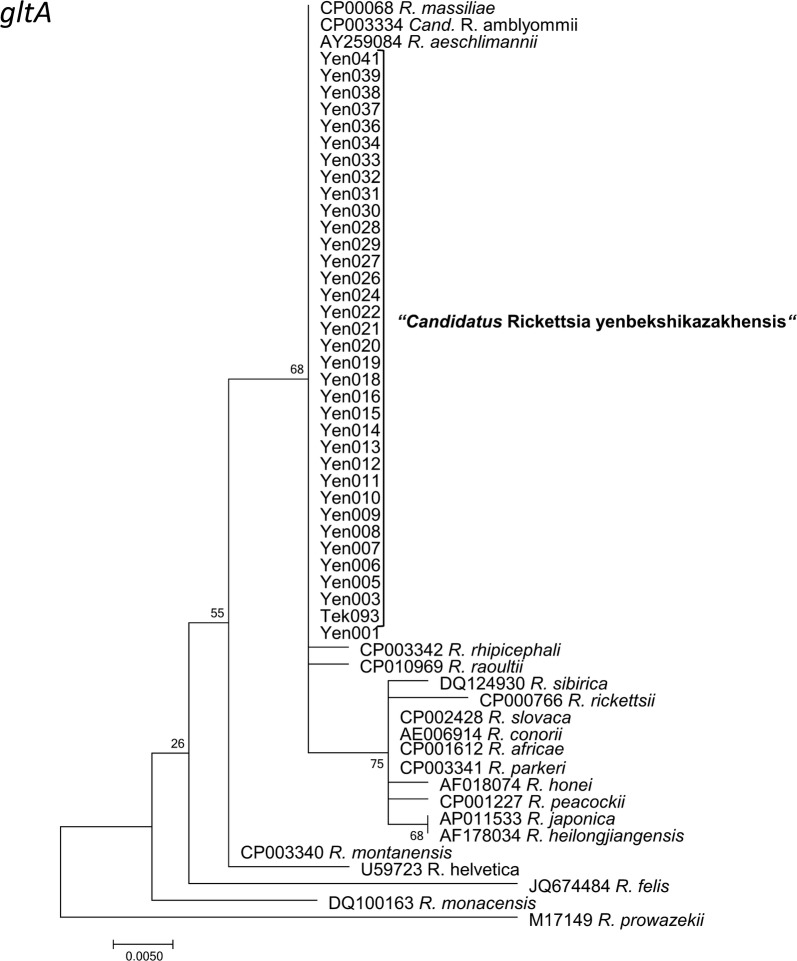
Maximum Likelihood phylogenetic tree based on partial 55 partial *gltA* sequences, with 35 sequences originating from Kazakh tick DNAs forming the new “*Candidatus* Rickettsia yenbekshikazakhensis” (34 from Yenbekshikazakh district, 1 from Yeskeldy district around Tekeli city) and 20 from GenBank. There were a total of 318 positions in the final dataset. The tree with the highest log-likelihood (-641.7358) is shown

As a result of MLST, BLAST and phylogenetic analyses, rickettsiae could be determined to the species level for 209 of the 212 *Rickettsia*-positive samples. A total of four *Rickettsia* spp. were identified in the molecular study (Tables 4, 5, 6, Figs. 2, 3, 4, 5, 6, 7, 8). Two already known species, *R. raoultii* and *R. slovaca*, were identified. *Rickettsia raoultii* was confirmed in 124 samples (124/209; 59.3%; 95% CI: 52.3–66.1%) by sequencing partial *ompB* (*n* = 123), partial *ompA*IV (*n* = 9) and 23S-5S (*n* = 9), in *D. marginatus*, *D. reticulatus* and *Hy. asiaticum* from Kyzylorda region (*n* = 113) and in the vicinities of Tekeli city in Almaty region (*n* = 11). Further, *R. slovaca* (*n* = 3) by partial *ompB* (*n* = 2), partial *ompA*IV (*n* = 1), 23S-5S (*n* = 2) genes was detected in *D. marginatus* pools only around Tekeli city in Almaty region (3/209; 1.4%; 95% CI: 0.3–4.1%), (Tables 4, 5, 6, Figs. 3, 4, 5, 6, 7, 8).

**Table 4:** Distribution of detected *Rickettsia* spp. in the tick species

Tick species	*R. raoultii*	*R. slovaca*	“*Candidatus* R. yenbekshikazakhensis”	“Genotype R. talgarensis”	Total	No. of tick pools
*I. persulcatus*	0	0	0	3	3	243
*H. punctata*	0	0	80	0	80	104
*D. marginatus*	119	3	0	0	122	129
*D. reticulatus*	3	0	0	0	3	3
*Hy. asiaticum*	1	0	0	0	1	17
*Rh. turanicus*	0	0	0	0	0	5
Total	123	3	80	3	209	501

*Note*: For three samples no sequences were obtained

**Table 5:** Distribution of *Rickettsia* spp. in the collecting localities

Locality	*R. raoultii*	*R. slovaca*	“*Canidatus* R. yenbekshikazakhensis”	“Genotype R. talgarensis”	Total	No. of tick pools
Almaty region						
Talgar district	0	0	0	2	2	104
Yeskeldy district (Tekeli city)	11	3	2	0	16	148
Yenbekshikazakh district	0	0	78	1	79	113
Kyzylorda region						
Syrdarya district	45	0	0	0	45	46
Shieli district	42	0	0	0	42^a^	46
Zhanakorgan district	25	0	0	0	25	44
Total	123	3	80	3	209	501

^a^For three samples no sequences were obtained

**Table 6:** Sequences with 100% homology to known *Rickettsia* spp.

*Rickettsia* spp.	*ompB*	*ompA*IV	23S-5S	*sca4*	*16S*	*gltA*	Total
*R. raoultii*	123	9	9	nd	nd	nd	141
*R. slovaca*	2	1	2	nd	nd	nd	5
Total	125	10	11	nd	nd	nd	146

*Abbreviation*: nd, not determined as rickettsiae could be identified by sequences of other gene fragments

The new “*Candidatus* R. yenbekshikazakhensis” was confirmed in 80 samples (80/209; 38.2%; 95% CI: 31.7–45.2%) and by MLST of the partial fragments of *ompB* (*n* = 77), *ompA*IV (*n* = 30), 23S-5S (*n* = 9), *sca4* (*n* = 34) and *16S* (*n* = 6), *gltA* (*n* = 35) genes (Tables 4, 5, 6, 7). “*Candidatus* Rickettsia yenbekshikazakhensis” was identified in 16.0% of all 501 tick pools studied (80/501; 95% CI: 12.9–19.5%). This rickettsia was detected with a high prevalence in *H*. *punctata* pools (80/104, 76.9%) from Yenbekshikazakh district (*n* = 78) and in the vicinities of Tekeli city (*n* = 1) in Almaty region, respectively. This “*Candidatus*” species was detected only in one tick species (*H. punctata*) collected from two districts (Yeskeldy and Yenbekshikazakh districts) of Almaty region (Tables 4, 5, 7, Figs. 3, 4, 5, 6, 7, 8).

**Table 7:** Overview of closest nucleotide identities of “*Candidatus* R. yenbekshikazakhensis” and “genotype R. talgarensis” with the first hit in BLAST with *Rickettsia* spp.

Gene	Maximum identity to known *Rickettsia* spp.	“*Candidatus* R. yenbekshikazakhensis”	Genotype R. talgarensis
*ompB*	> 99.2	99.0% (CP013133): *R. rhipicephali*	na
*ompA*IV	> 98.8	99.0% (U83446): *R. aeschlimanni*	92.7% (CP003304): *R. canadensis*
23S-5S	na	96.1% (AY125016): *R. aeschlimannii*	88.4% (CP003304): *R. canadensis*
*16S*	> 99.8	99.8% (CP003319): *R. massiliae*; 99.8% (HM050274): *R. aeschlimannii*	99.4% (CP003319.1): *R. massiliae*
*sca4*	> 99.3	99.1% (HM050275): *R. aeschlimannii*^a^; 98.3% (HM050275): *R.aeschlimannii*^b^	na
*gltA*	> 99.9	100% (CP015012): *R. amblyommatis*; 100% (KU723495): *R. aeschlimannii*; 100% (KT588058): *R. massiliae*	na

* According to [8]

^a^All sequences from Yenbenshikazakh

^b^Sample Tekeli 093

*Abbreviation*: na, no sequences available for comparison

We further detected the new “genotype R. talgarensis” in three samples by analysis of the partial *ompA*IV (*n* = 3), 23S-5S (*n* = 2) and *16S* (*n* = 2) genes in the ticks (3/209, 1.5%) in 0.6% of all 501 tested tick pools (3/501; 95% CI: 0.1–1.7%) (*n* = 2341). This genotype was only present in *I. persulcatus* collected in two districts (Talgar and Yenbekshikazakh) in Almaty region (Tables 4, 5, 7, Figs. 3, 4, 5, 6, 7, 8).

Unfortunately, one sample could not be sequenced and in two samples a mixture of different *Rickettsia* species was detected by sequencing of gene fragments. Overlapping chromatograms indicating a mixture of sequences were found for partial *ompA*IV, *gltA*, *16S* and *sca4* sequences for sample Kyzylorda 061 (*D. marginatus*, Kyzylorda region, Shieli district) and in the *ompB*, *ompA*IV and *gltA* sequences for sample Tekeli 076 (*D. marginatus*, Almaty region, Yeskeldy district), respectively.

## Discussion

To our knowledge, this study is the first large-scale, comprehensive investigation of rickettsiae of the spotted fever group conducted in two selected pilot regions of Kazakhstan. The difference in natural landscapes in both selected regions explains the variety of collected ticks. *Dermacentor marginatus* is the most abundant tick typically found at the collection sites in the desert and semi-desert landscape of Kyzylorda region [26] which mirrors the habitat of this tick species [46, 47]. In comparison, the three selected collection sites in the Almaty region are characterized by the presence of a mountainous landscape covered with forests which are the classical habitats for *Ixodes* spp. [46] exhibiting in Almaty region the highest abundance of all tick species (48.1%). Almaty region showed the wider variety of tick species with five out of the seven species identified in this study (*I. persulcatus*, *H. punctata*, *D. marginatus* and *D. reticulatus*).

The identification of the ticks investigated in this study was performed using morphological markers [27–30]. For *D. marginatus* and *D. niveus*, there is an ongoing discussion if these two species are conspecific. Genetic markes seem to give evidence for that despite a detailed comparison is still missing [48–50]. Herein, both morphologically different species were summed up and data presented as data for *Dermacentor marginatus.*

Our results show that five of the seven collected tick species are positive for *Rickettsia* spp. In general, in Kyzylorda region where *Dermacentor* spp. dominated, 56.8–100% of the ticks’ pools were *Rickettsia*-positive, and only *R. raoultii* was found in the two species of *Dermacentor*. Surprisingly at the three collection sites in the Almaty region, which has been considered so far as a non-endemic region, all four *Rickettsia* species detected in this study were found. *Rickettsia raoultii* was detected in 59% of the tick pools and *R. slovaca* was detected in three pools; both species are human pathogens. The present data indicate that the main vectors of these two pathogens are ticks of the genus *Dermacentor*, which is in line with data from neighboring countries, i.e. Russian Federation, Mongolia or northwestern China which is located close to the Almaty region of Kazakhstan [7, 51–59]. Of note, in our study *R. raoultii* was also for the first time detected in one *Hy. asiaticum* tick pool collected from Kyzylorda region of Kazakhstan.

*Rickettsia. raoultii* and *R. slovaca* are known human pathogens that cause the scalp eschar and neck lymph adenopathy after a tick bite (SENLAT), tick-borne lymphadenopathy (TIBOLA) or *Dermacentor*-borne necrosis erythema lymphadenopathy (DEBONEL) after a tick bite [7, 60]. The high MIR of *R. raoultii* in the ticks studied and a recent case study in China were 26 cases of *R. raoultii* infections with varying severity were described [61], indicate that *R. raoultii* should be included in the diagnosis of rickettsioses in Kazakhstan. The occurrence of *R. slovaca* was previously described in *Melophagus ovinus*, the sheep ked, collected in localities of Xinjiang Uygur Autonomous Region (northwestern China), that borders the Almaty region of Kazakhstan [58]. The detection of *R. slovaca* leads to the conclusion that further data on its natural foci in Kazakhstan as well as the role for human infections are needed.

We here report a new “*Candidatus* R. yenbekshikazakhensis” by performing a MLST of six gene fragments. For the *ompB*, 23S-5S, *16S* and *sca4* but not for the *ompAIV* and *gltA* it fulfills the criteria of Fournier et al. [8] to designate it as a new “*Candidatus*” species (Table 7). It has been suggested to taxonomically classify rickettsiae as new “*Candidatus*” if at least four or five sequences are newly described [4, 8–10]. The closest species is *R. massiliae* which is also known to be pathogenic to humans inducing a SENLAT syndrome [62, 63]. The new “*Candidatus* R. yenbekshikazakhensis” was detected in two regions and in 87.6% of all *H. punctata* ticks studied, which might therefore be its main vector.

Further, the “genotype R. talgarensis” was detected in three tick pools. The analysis of three gene fragments, *ompAIV*, 23S-5S and *16S* could be performed showing a quite high divergence to all known rickettsiae (Table 7). The detected agent fulfills therefore, the criteria to be described as a new genotype [8]. For both, “*Candidatus* R. yenbekshikazakhensis” and “genotype R. talgarensis” the pathogenicity is still unknown and should be the aim of further studies.

## Conclusions

The clinical cases of tick-borne rickettsioses, which were registered by using CFT over the past 20 years in Kazakhstan, are so far not confirmed by other serological methods such as ELISA and by pathogen detection (e.g. rickettsial DNA by PCR). With the rising evidence on the relevance of rickettsiae in human infections and for improving epidemiological data, routine laboratory diagnostic tools must be implemented in all reporting laboratories in Kazakhstan. Our data also indicate that clinicians should be aware of SENLAT syndrome which is caused by two confirmed pathogens (*R. raoultii* and *R. slovaca*) circulating in the territory of Almaty and Kyzylorda regions. The present data indicate that tick-borne rickettsiae and associated pathological conditions in humans should be further investigated in all regions of Kazakhstan to estimate the importance and clinical impact caused by all four described rickettsiae.

## Abbreviations

CFT: complement fixation test
DEBONEL: *Dermacentor*-borne necrosis erythema lymphadenopathy
MCL: maximum composite likelihood
MIR: minimum infection rate
MLST: multilocus sequence typing
PCR: polymerase chain reaction
SENLAT: scalp eschars and neck lymphadenopathy
TIBOLA: tick-borne lymphadenopathy
